# Highly Efficient Lossless Coding for High Dynamic Range Red, Clear, Clear, Clear Image Sensors

**DOI:** 10.3390/s21020653

**Published:** 2021-01-19

**Authors:** Paweł Pawłowski, Karol Piniarski, Adam Dąbrowski

**Affiliations:** Division of Signal Processing and Electronic Systems, Institute of Automation and Robotics, Poznań University of Technology, Jana Pawła 24, 60-965 Poznań, Poland; karol.piniarski@put.poznan.pl (K.P.); adam.dabrowski@put.poznan.pl (A.D.)

**Keywords:** lossless video compression, RCCC image sensor, HDR, ADAS, ADS, automotive systems

## Abstract

In this paper we present a highly efficient coding procedure, specially designed and dedicated to operate with high dynamic range (HDR) RCCC (red, clear, clear, clear) image sensors used mainly in advanced driver-assistance systems (ADAS) and autonomous driving systems (ADS). The coding procedure can be used for a lossless reduction of data volume under developing and testing of video processing algorithms, e.g., in software in-the-loop (SiL) or hardware in-the-loop (HiL) conditions. Therefore, it was designed to achieve both: the state-of-the-art compression ratios and real-time compression feasibility. In tests we utilized FFV1 lossless codec and proved efficiency of up to 81 fps (frames per second) for compression and 87 fps for decompression performed on a single Intel i7 CPU.

## 1. Introduction

In the last decade we see a significant acceleration in the development of camera-based advanced driver-assistance systems (ADAS) for the automotive industry. So far limited to luxury automobiles, ADAS technology is now becoming also popular in the standard segment of cars. Typical ADAS facilities help drivers in several aspects: automatic parking [1], surround-view, reverse driving, avoiding lane departure with detection of driver tiredness. Moreover, they offer many safety critical facilities like pedestrian detection, road sign detection automatic braking [2,3], and many others. Most of the ADAS solutions collect information about the car surroundings using cameras.

The camera-based solutions are also under development in autonomous driving systems (ADS) which are related to semi-autonomous and autonomous vehicles. The data from the cameras need to be stored for safety reasons (e.g., for an after accident investigation) or for improving algorithms accuracy in the laboratory conditions, typically in the software in-the-loop (SiL) or hardware in-the-loop (HiL) tests [4]. 

Nowadays an automatic or even remote vehicle control is also considered in emergency situations before achieving the state of the fully autonomous cars equipped with ADS [5,6]. In consequence, many camera-based ADAS and ADS solutions produce huge amounts of data that need to be stored onboard or transferred via a wireless network.

To reduce this huge amount of data appropriate data compression algorithms should be developed. Currently, most of automotive applications, mainly due to the broadly understood safety, do not allow any lossy data compression (the processed data must be identical to the source data). Unfortunately, lossless compression techniques cannot fulfill the need of high compression ratio [7,8]. Even the compressed data (especially this losslessly compressed) is a huge problem both for onboard storage and for wireless transmission [9] (the high throughput of the 5G wireless transmission standard is surely needed in this case, however it is still in a preliminary stage in the automotive field [10]).

In automotive applications, the high dynamic range (HDR) video format must be used to obtain high quality images. Without the HDR it is not possible to guarantee the image quality in various lighting conditions, e.g.: very low light at night, or the full light of sunny days, or in the darkness with high light reflections [11].

In recent years, in contrast to typical red, green, blue (RGB), or more strictly speaking the red, green, green, blue (RGGB) Bayer mosaic, image sensors, a new red, clear, clear, clear (RCCC) image sensor has been proposed for the automotive industry. The RCCC sensor, similar to a monochrome sensor, is more sensitive, especially in low light conditions, offers better reproduction of details, but still provides the separate red color information [12].

The interpolated grayscale image (from clear pixels) is used for automatic detection purposes like e.g., detection of cars, pedestrians, obstacles, or traffic signs. On the other hand, the red channel is used for detection of vehicle backlights, road signs, and traffic lights [13,14].

Because a new class of the HDR RCCC cameras internally compress the HDR data to reduce the number of bits per pixel, but still produce more than 8 bit per pixel, a compression of such video stream becomes a quite new, non-typical problem. In consequence, a new data compression task arises, namely efficient lossless compression of HDR RCCC video sequences for ADAS and ADS applications. Therefore, in this paper we consider and propose a new lossless solution for this task in order to simultaneously achieve: high compression ratio, high dynamic range, and high computational efficiency.

The paper is structured as follows. In the next section we introduce lossless video data compression requirements for ADAS and ADS applications and define the proper requirements for the lossless codec. Then, we describe the new class of HDR image sensors and color filter arrays. In In Section 4 we present the state-of-the-art in the lossless image compression. In Section 5 we analyze the RCCC format features, possibilities of its compression and we propose four various divisions of RCCC components before the compression. In Section 6 the experiments are presented with the FFV1 lossless codec using experimental ADAS/ADS dataset, together with the multi-threaded implementation of the best coding procedures. Finally, we prove that the proposed procedures for RCCC codecs enable the real-time compression and decompression with the state-of-the-art compression ratios, using a single CPU only. Several important conclusions close the paper.

## 2. Lossless Compression for ADAS and ADS

The amount of data registered with the cameras in ADAS and in ADS is huge. For example, one 4K (4096’2160 pixels) grayscale camera with 12-bit per pixel image sensor and 30 fps transfers ca. 400 MB/s uncompressed data. Logging 8 h of recordings taken from only four cameras needs more than 46 TB of storage space. In consequence, real-time transmission or logging of such huge amounts of data is a serious problem. Thus, it is necessary to use severe data compression.

As it was mentioned in the Introduction, the data from the ADAS/ADS cameras needs to be stored for safety reasons, e.g., for an after accident investigation and for developing or testing safety algorithms in the laboratory (SiL or HiL systems) [4]. Mostly lossless codecs are used as large certainty of object detection is the most important parameter for safety reasons. However, the lossless compression ratio remains low (e.g., for RGB 24 bpp images it does not exceed 3.3 [15]). In ADAS/ADS applications, compression must be performed in real time by an on-board computer system with limited capacity, which precludes (at least currently) the use of complex compression algorithms, such as deep neural networks (DNN) [16,17].

For less critical automatic video processing applications, e.g., the remote vehicle control in emergency situations a lossy compression of the registered data is possible [5,6]. However, such data loss can substantially affect accuracy of the automatic video processing algorithms [18,19,20].

The important task of the ADAS/ADS designer is to select and adopt right codec for a given application. Regardless lossy or lossless codec is chosen, the requirements for automatic detection systems are the same: real-time processing, high compression ratio, and high and stable quality of the compressed images. Of course, the highest possible quality is ensured by the lossless compression but in cost of a relatively low compression ratio. Consequently, in this paper we propose lossless codec procedures prepared for the RCCC image sensors, one of the most important standard in the ADAS/ADS.

## 3. New Class of HDR Image Sensors

Technical requirements regarding the image sensors that are embedded in cameras for automotive industry are very high. The most important requirement is related to the high quality of the recorded images (in various conditions e.g., in very low light at night, or in full light during sunny days, or in the darkness with high light reflections). The other requirements concern hard environmental conditions (variety of temperatures, vibrations), and high demands of the reliability, safety integrity levels and limited production costs [21]. In fact, very similar requirements exist in the mobile phone market, so the interest in such products is very large and growing very fast.

### 3.1. High-Dynamic Range Image Sensors

Camera manufacturers have already reacted to these market needs. In recent years several new types of camera sensors have been proposed. Some manufacturers (e.g., STMicroelectronics, On Semiconductor) offer special HDR image sensors They are also known as wide dynamic range (WDR) sensors since they produce images with the dynamic range up to 132 dB or 22 bits [22,23]. Such ultra-high values are reached using different techniques including multiple exposures or split pixel technology supported by tone mapping and automatic HDR image processing even inside the imaging chip [2,22,23,24]. Using the multiple exposure technique the HDR image data is constructed by combination of three exposures (integration time) for each pixel. A long exposure captures details in the dark parts of the scene, a short exposure captures details in the bright parts, whilst a mid-length exposure captures all mid-range details [25]. As soon as a pixel’s three exposure values are available, they are combined to create a linearized HDR value for each pixel’s response.

**Figure 1:**
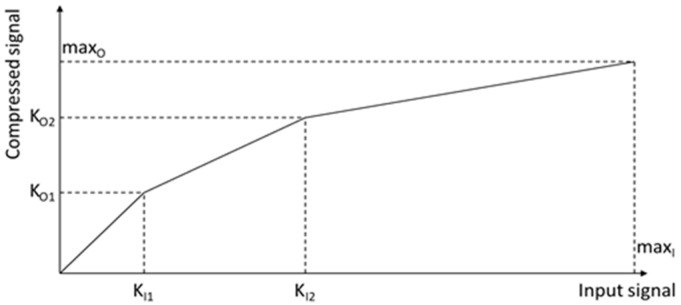
Piecewise linear representation in HDR image sensors.

Unfortunately, the HDR image sensor produces much more data than the standard dynamic range (SDR) sensor. A simple piecewise linear (PWL) compression (a logarithmic type compression) is typically used to compress the bit-length of image data (Figure 1). Input signals from the image sensors, with values up to the first knee point ($K_{I1}$) are not changed. Signal values greater than $K_{I1}$, but lower than $K_{I2}$ are compressed by reduction of the number of bits that represent an excess value (between $K_{O1}$ and $K_{O2}$. After each knee point further bit reduction for next excess values arises. The higher the value, the more compressed it is. In consequence, high values of the signal (very bright pixels) are represented with lower accuracy, but the image sensor produces HDR images with a relatively low bits per pixel number.

This compression is similar to the Drago tone mapping algorithm for HDR images [25]. In the HDR image sensors this typically reduces data words by 10 bits (e.g., from 22 bits to 12 bits) while still preserving the image details. It also reduces requirements for the bandwidth during the video stream transmission from the image sensor to the video processor. Notice, that in high resolution sensors, even reduced bandwidth is still very high, e.g., for 4K video (4096´2160 pixels), 30 fps frames per second, 12 bpp it is ca. 3.2 Gb/s.

Because the output data length is higher than the number of bits generated by the image sensor in each exposure, the PWL compression may be lossless. In [23] the producer ensures that the compression of 20-bit HDR image to 14-bit is lossless, while compression to 12-bit introduces a minimal data loss. The other producer [22] claims that the PWL compression algorithm is lossless in the sense that any losses in the image quality are below the noise floor of the image sensor. The number and values of knee points in PWL compression may be fixed (like in Figure 1, where the number of knee points is 2) or user-defined, even up to 32 points to: improve SNR performance, reduce quantization noise, and produce more natural images [22,23].

The compression reduces the amount of video data that should be transferred from the image sensor. It is very desirable, especially in mobile, low-power and low-cost solutions. The problem of this transfer was noticed and standardized as the camera serial interface (CSI) standard by the Mobile Industry Processor Interface (MIPI) Alliance [26]. Although the CSI standard allows direct transmission of HDR images (e.g., with a length of 20 bits), the HDR image sensors typically offer internal PWL compression to 12 or 14 bits [22,23].

### 3.2. Novel Color Optical Filter Arrays

The image sensors for automotive cameras differ from typical cameras not only in the dynamic range, but also in the color mosaic (also called the color optical filter array).

**Figure 2:**
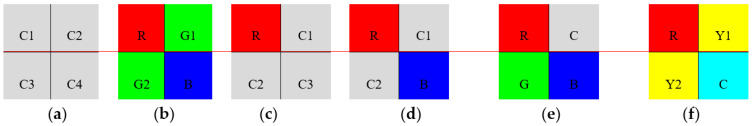
Color filter arrays: (**a**) monochrome (CCCC), (**b**) RGGB, (**c**) RCCC, (**d**) RCCB, (**e**) RGBC, (**f**) RYYC.

Six optical color filter arrays that may be found in ADAS and ADS solutions are presented in Figure 2 as 2 × 2 periodic patterns that constitute the whole imaging sensor.

After the simplest monochrome sensor (that without the color filters, Figure 2a), the most typical color filter array is the so called Bayer filter with red, green, green, blue components (RGGB, Figure 2b). One red, one blue and doubled green elements offer similar sensitivity of the human eye and a cost-effective image sensor technical solution with good reproduction of colors. Unfortunately, R, G, and B filters reduce the amount of light reaching the respective sensors by about 2/3, compared to a monochrome sensor [27]. Additionally, to obtain the full resolution colorful image an interpolation is needed to compute missing color components for all pixels. This procedure can produce false colors, especially on lattice-like areas.

The above disadvantages are very undesirable in ADAS and ADS applications. Therefore, instead of the Bayer color filter, the RCCC color filter is commonly used. The RCCC consists of red, clear, clear, clear sensors (clear means colorless, i.e., fully transparent or more precisely panchromatic filter, i.e., transmitting all visible spectral colors), see Figure 2c. Unlike the RGGB Bayer sensor, the RCCC sensor uses only the red color optical filter, and for each four horizontally/vertically neighboring pixels in the mosaic three of them have clear filters. Hence, the image generated by the RCCC sensor is almost as detailed and bright as the monochrome sensor but still provides the red color component information [11]. This improves ability of automatic detection of traffic lights, rear lights of vehicles, important informative parts of road signs, etc.

Having RGB components for every pixel, luminance (the monochrome image component) can be easily calculated. In the case of sensors with C (clear) elements the luminance may be directly defined as the C component. Thus, with the RCCC sensor 3/4 of all luminance values are directly measured, and 1/4 of them only (in places of R component) are missing and must be interpolated. Therefore, in contrast to images registered with the Bayer RGGB mosaic, the RCCC offers much better representation of details (higher relative resolution) and less noise, especially for low lightning conditions [4,13,14]. Unfortunately, an attempt to perfectly convert the RCCC to RGGB format is impossible, as there is no information about the relationship between the missing G and B components in the RCCC format.

There are also other mosaics used, shown in Figure 2: red, green, blue, clear (RGBC), red, clear, clear, blue (RCCB), red, yellow, yellow, cyan (RYYC), or red, green, blue, clear (RGBC), but currently, mainly due to their lesser sensitivity and lower relative resolution, they are much less popular than the RCCC mosaic in ADAS and ADS applications. Please note that these notations may be confusing. In RCCC C means clear but in RYYC, or CMY (cyan, magenta, yellow) it means cyan. Here Y means yellow but it also may mean luminance. Regardless, the lossless data compression solutions developed in this paper for the RCCC mosaic may also be used for other color filter mosaics.

## 4. State-of-The-Art in the Lossless Compression of Images

A comprehensive but very deeply investigated survey of the image compression techniques, both lossless and lossy is presented in [28]. Unfortunately, in contrast to lossy compression methods, there are only a few main techniques for lossless compression. The best known of them are the classic Huffman coding, arithmetic coding and predictive coding [28]. Taking into account the properties of the dataset under compression, the main method for compression of an image is prediction. This may be performed using neighboring pixels, due to their similarity within the image. This is called the intra-frame prediction. For this purpose, the image is usually analyzed from the top-left to the bottom-right corner [15].

In the motion video there are additional similarities to be used, i.e., those between the same pixels in consecutive video frames (both previous and/or subsequent ones). This is called the inter-frame prediction [28]. From the very beginning of the lossy video compression history, this is the main compression mechanism, which allows to achieve high compression ratios. Unfortunately, such mechanism must include the motion prediction but computationally this is very complex [28].

In practice, most of the lossless video codecs use the intra-frame compression only due to the fact that in the lossless mode they achieve similar compression ratios to that of the inter-frame compression, while the inter-frame prediction is much more complicated.

The reference pixel value together with the prediction errors are only transmitted to the decoder [15,28]. In order to restore original data, the same prediction mechanism is used in the decoder.

One of the best known lossless compression standard is JPEG-LS, but even in the newest mainstream lossy standards like the high efficiency video coding (HEVC) there are some modes or ideas that can be used in the lossless compression [29]. HEVC was originally designed for lossy video compression, and thus is not ideal for lossless video compression. In paper [30], the authors propose an efficient residual data coding method for HEVC lossless video compression. Authors of some articles e.g., [31] propose lossless compression to eliminate statistical redundancy in the coded video bit stream.

An area where the lossless compression is often used is medicine. The authors of [32] compared the performance of five lossless video codecs, i.e., H264, H265, Lagarith, MSU, MLC and three still-image codecs, i.e., JPEG, JPEG2000, JPEG-LS using 3D medical computed tomography datasets. Unfortunately, the results are not very valuable for the ADAS/ADS applications, as while the medicine applications do not put strong limitations on the computational complexity of the solutions, they typically do not require real-time operation.

DNNs became a very popular solution for performing various classification, prediction, and identification tasks in images, including also image compression [16,33]. A recent overview of the research in the field of video compression using DNNs is presented in [17]. The authors of this survey point out two main directions of research in this area: improving existing video codecs by performing better predictions, and the elaboration of holistic methods for the end-to-end image or video compression schemes. They conclude that while some of the results are promising, no breakthrough has been reported so far [17]. It is worth noting here that the methods based on DNNs are still too complicated to operate in ADAS/ADS applications in real-time with the limited capacity of the on-board computing systems used.

Almost all image compression research works involve compressing monochrome or color images in the RGB format. However, the considered RCCC compression, to achieve relatively high compression rates, requires a quite different approach. However, a somehow similar approach to that presented in this paper was used in several papers, which propose a lossless compression dedicated to sensors with the RGGB mask (cf. Figure 2b). The authors of [34] present a survey on lossless compression of such RGGB (Bayer pattern) color filter array images. They also propose to split the image into three new images corresponding to each channel (R, G, and B) and study the same compression algorithms applied to each of them individually. This allows an improvement of more than 15% in prediction based methods [34]. Also in [35] the authors evaluate the performance of some methods for lossless and near-lossless compression for the real raw Bayer pattern.

Reference [36] is very close to our ADAS/ADS application. In high-speed applications, or applications, in which the system design requires low-latency (low-complexity compression), the new JPEG XS standard offers a solution to compress Bayer pattern images close to the video sensor, while maintaining visually lossless quality. This paper presents contributions to JPEG XS that are currently under the discussion in the JPEG committee (SC29WG1) [36]. Unfortunately, this is not the perfectly lossless solution.

To perform compression of HDR images (a typical task for ADAS/ADS), we just have to assume that the lossless video codec used must support high dynamics video. In our previous work [15] we already tested most important codecs for ADAS, however, with the thermo-vision recordings only and not using the RCCC standard. The nature of thermal images is more similar to monochrome images and does not introduce problems such as in the RCCC format, which is the main subject of this paper. We found that for both: lossless and lossy compression with proper codecs, a possibility exists for achieving real-time performance with a single CPU. The best codec that exceptionally achieves both high compression ratio and real-time performance for lossless compression occurred to be the open-source FFmpeg FFV1 codec [37,38]. It is a high quality codec, that achieves the state-of-the-art compression ratios around 3.3 for 24 bpp RGGB images (with 8 bit per one color component), but it also supports a 16-bit (per component or per pixel) HDR image format [15]. Thus it is also chosen in the present work to perform experiments.

Beside the advantages presented above, the FFV1 codec offers a very valuable option. It uses the inter-frame prediction only, but by setting the size of the group of pictures (GOP) higher than 1 it builds broader context model for coding the predictor errors. The model is calculated not only inside one frame, but using many frames, i.e., those inside the GOP. It results in a slightly increased compression ratio due to better fit of the model to the content of the used frames. Unfortunately, such broad context model disables a possibility of independent (parallel) processing of consecutive images [37].

To summarize, in lossless coders for ADAS and ADS real-time applications, to reduce the computational complexity, only very simple predictors can be used, which merely utilize similarity of neighboring pixels in one frame. For example, the predicted pixel can be computed as a simple copy of the neighboring one, or as a mean or a median of the neighboring ones.

The more information the predictor takes into account, the more accurately it can predict and the prediction errors to be encoded are smaller. Thus, low complexity of the predictor mechanism (and consequently the encoder) results in a high computational efficiency of the decoder [15,28].

Concluding, the more similar the neighboring pixels, the better are the compression results. Of course, they mainly depend on the image content, but not only. In the next section, we propose how to improve, by a proper manipulation of pixels, the compression results using the best state-of-the-art lossless image codecs.

## 5. Proposal of Four Procedures for Lossless Compression of Specific HDR RCCC Image Format

**Figure 3:**
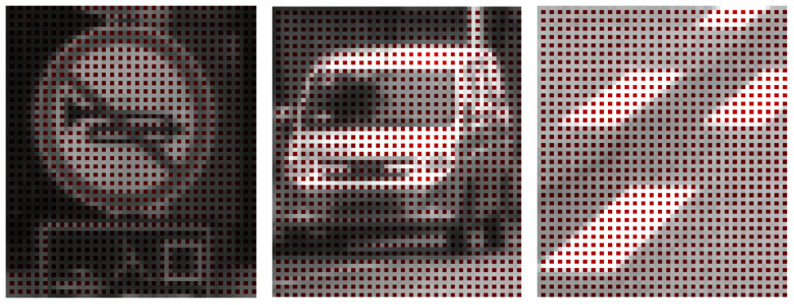
Three sample parts of source RCCC image.

**Figure 4:**
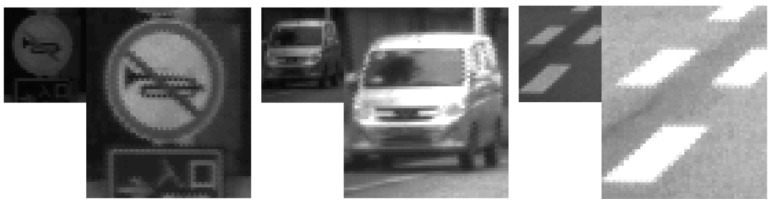
Three sample parts of source RCCC image decomposed into two images with R and CCCC components (the second image is created with the CCC components while the missing values are interpolated).

**Figure 5:**
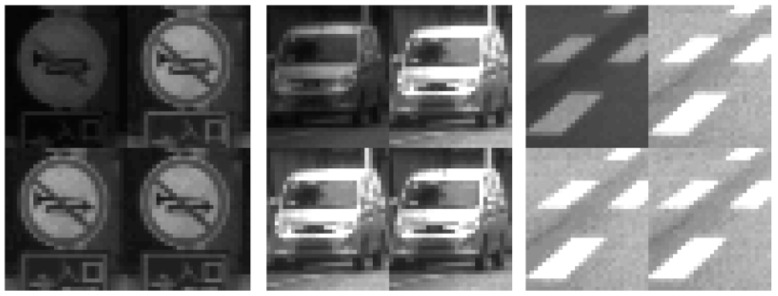
Three sample parts of source RCCC image decomposed into four images with all components R, C1, C2, C3 separated.

**Figure 6:**
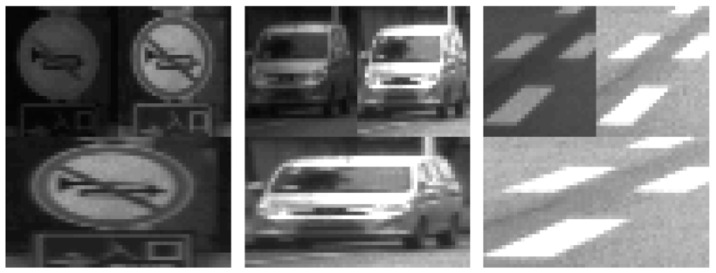
Three sample parts of source RCCC image decomposed into three images: two small images with R and C1 components and a horizontally two times larger image comprising C2 and C3 components.

According to the best of the authors’ knowledge, up to now, there is no special image or video codec proposed that directly and losslessly compresses the RCCC image format. Therefore, by the RCCC codec procedure we mean an interface that makes the effective compression of the RCCC format data possible with standard lossless codecs (those originally thought to work with grayscale or typical color video formats). Consequently in this paper, we propose (four) following procedures of using standard image codecs for compression (encoding) the RCCC images with various divisions of components:

- First, RCCC image may be treated as a monochrome image and encoded directly (see Figure 3). Notice that the R component is spatially sub-sampled (consecutive R values do not occur in the direct neighborhood) and the difference between R and C components may be significant. Certainly, the same phenomenon is also valid for conventional video formats [20]. This reduces accuracy of prediction based on neighboring pixels. Horizontal neighborhood of C2 and C3 components (see Figure 2c) is closer than the vertical one, thus the prediction using the typical prediction masks is less accurate.
- Second, due to the informative differences between R and C components and, in consequence, expected errors in their joint prediction, R and CCC components can be decomposed into two separate images. Resolution of the image created with R components only is certainly ¼ of the resolution of the source RCCC image. We suggest that in the second image, created from the CCC components, the missing values (i.e., those previously occupied with R component values) should interpolated, resulting in the CCCC image preserving the original resolution (see Figure 4). In this method, the encoder has by 25% more data to encode than in the previous method, but inside the decomposed images the pixels are more similar in the neighborhood and the overall compression can be more effective. There are various ways possible to interpolate the missing C values. In order to maximize the compression ratio we propose to calculate them just with the predictor. In this way, the prediction errors are equal to zero for the interpolated C values.
- Third, all four components, i.e., R, C1, C2, C3 can be decomposed into four separate images with equal resolutions of ¼ of the source RCCC image resolution (see Figure 5). Compression of single components results in small prediction errors, but we lose information about the closest neighborhood between components C2 and C3. However, this method is the most regular and universal as it can be directly used to any color filter mosaic not only to RCCC (cf. Figure 2).
- Last but not least, components of the RCCC source image can be decomposed into three images: two small images R, C1 (both are just the same as in the previously described possibility) and to a horizontally two times larger image comprising C2 and C3 components (see Figure 6). Compression of C2 and C3 components together, due to their direct proximity, may be more efficient than the separate one.

To choose the best compression procedure and check the compression ratios and the real-time performance achievable with the proposed lossless compression procedures we performed a set of experiments with real ADAS/ADS video sequences using a modern FFV1 codec.

## 6. Experiments on Lossless HDR RCCC Codec and Implementation of Coding Procedure

To decide which of the presented above divisions of components and the related compression procedures is the best (in the meaning of compression ratios and compression/decompression throughputs) we check it experimentally with the FFV1 lossless codec. The experiments we performed with real ADAS/ADS recordings.

### 6.1. Methodology

The following metrics were used to evaluate the effectiveness of the proposed video coding procedures of the RCCC format including: compression ratio, performance, and speed of operations [39]:

- compression ratio:
  (1) $$\mathrm{CR}=\frac{\mathrm{size}\;\mathrm{of}\;\mathrm{uncompressed}\;\mathrm{stream}}{\mathrm{size}\;\mathrm{of}\;\mathrm{compressed}\;\mathrm{stream}}$$
- compression throughput of the input stream:
  (2) $${\mathrm{Thr}}_{\mathrm{in}}=\frac{\mathrm{size}\;\mathrm{of}\;\mathrm{uncompressed}\;\mathrm{stream}}{\mathrm{compression}\;\mathrm{time}}\left[\mathrm{MB}/s\right]$$
- decompression throughput of the output stream:
  (3) $${\mathrm{Thr}}_{\mathrm{out}}=\frac{\mathrm{size}\;\mathrm{of}\;\mathrm{decompressed}\;\mathrm{stream}}{\mathrm{decompression}\;\mathrm{time}}\left[\mathrm{MB}/s\right]$$

The introduced distinction between the throughputs at the input and output makes the analysis of the compression and decompression speeds possible and does not depend on the compression ratio.

Additionally, we calculated the throughputs of compression or decompression as ratios of numbers of the processed frames to the compression or decompression time. By this means the throughput is also expressed in frames per second (fps).

### 6.2. Lossless Video Codec

As we mentioned in Section 4, for experiments we decided to use the state-of-the-art FFmpeg FFV1 codec [37,38]. All decomposition strategies of RCCC images were tested in both compression modes which this codec offers, i.e., the standard mode of compression (i.e., that without building the context model) and the extended mode of compression (i.e., that with the context model that is built using GOP > 1). The standard mode was implemented with GOP equal to 1 and the extended mode with GOP equal to 100. The latter GOP value was chosen as the best setting of this mode, after a series of experiments.

### 6.3. RCCC Video Database

For all experiments 11 uncompressed video sequences of 12 bpp/36 fps RCCC format have been used. These sequences have been registered by HDR RCCC camera mounted in the front of the car. The sequences are typical for ADAS and ADS applications and include various road scenes, types of roads and environments, and various weather conditions. The average length of the sequence is about 1800 frames (50 s). Details about the sequences are presented in Table 1.

Although we used ADAS/ADS video sequences that contained 12 bpp, it should be noted that with these 12 bits HDR images were represented using the PWL compression. The introduction to the PWL compression in HDR cameras is given is Section 3.1.

In the tested ADAS/ADS sequences, the PWL compression was done by the camera’s internal processor with the following parameters: two knee points and three various, uniform quantization regions. The input knee points are: $K_{I1}=2048$, $K_{I2}=65,536$, and the output knee-pints are $K_{O1}=2048$ and $K_{O2}=3040,$ respectively (cf. Figure 1). It gives three quantization steps: the first one, up to the 11-th bit with the unit quantization step, the second one with the 64 times larger quantization step, i.e., the unit step shifted by 6 bits, and finally with the 1024 times larger quantization step, i.e., that shifted by additional 4 bits. Such compression covers the overall input HDR dynamic range of 11 + 6 + 4 = 21 bits.

Although our test database offers 21-bit per component HDR images, even after the PWL bit-length compression into 12 bits, it contains up to 4-bit noise level floor. This will reduce the compression ratio, but the presented experimental results will be very close to the real measurements.

Because the tested image codec (i.e., FFV1) does not support 12 bpp format, before the compression, all source 12-bit RCCC images were transformed into 16-bit RCCC images to match the input codec depth. This was done by storing 12-bit RCCC data in 16-bit words and setting 4 most significant bits to 0. This transformation did not affect the resulting compression ratio as we did not count these 4 zero bits into the input stream.

In fact, in lossless compression of HDR images, the PWL bit-length compression may be neglected (decompressed files are identical to those before compression, the designed codec supports the full 16-bit format).

However, it should be noted that the presented ideas are not limited to a specific number of bits (e.g., 12 bpp). This value comes from the ADAS recordings and is used just for experiments to show the real values. Apart from this fact, our solutions can be applied to image sensors with any dynamic range (expressed in bits), although the software, we have prepared, can compress images up to 16 bits.

### 6.4. Testbed and Experimental Setup

We assumed that the preferred processing platform to use in a vehicle is similar in performance to a standard computer with a single processor-based CPU. For all tests we used a mid-class Intel i7-3770 processor. This platform is cheap, compact, and achieves much lower power consumption in comparison to the general purpose computing on graphics processing unit (GPGPU) solution. However, in some applications, e.g., for simultaneous compression of several high-resolution streams (e.g., 4K), GPGPU or FPGA (field programmable gate array) platforms are needed. The codec implementation was written in C/C++ programming language using the FFV1 codec v.1.3 with the FFMPEG library.

### 6.5. Selecting the Best of Lossless RCCC Codec Procedures

In the beginning of the experiments we checked how the division of R and C components in RCCC images influences average compression ratios and the throughputs of compression and decompression. Results of averaged compression ratios and averaged throughputs are presented in Table 2 and Table 3. The CR, ${\mathrm{Thr}}_{\mathrm{in}}$, and ${\mathrm{Thr}}_{\mathrm{out}}$ values were averaged over all 11 video sequences (shown in Table 1).

We tested all four proposed strategies, namely: direct RCCC image, interpolated CCCC and R images, all four components (R, C, C, C) separated, and finally, separated R, C1 and joined C2 and C3 components (for details see Section 5).

Direct compression of RCCC image, i.e., without the component decomposition, although simple, gives clearly worse CR than the other methods (CR = 1.47 for GOP = 1 and CR = 1.50 for GOP = 100). This inefficiency is due to significant informative differences between R and CCC components and in consequence missed prediction between quite different, but placed next to each other in the image, R and C components with (cf. Figure 2c).

The coding of interpolated CCCC and separated R images performs better, but it is also less efficient that the other two methods with components separated (CR = 1.97 for GOP = 1 and CR = 2.04 for GOP = 100). Even with the correct prediction for each interpolated C value (one for group of four pixels, in the place of R component), the 0 prediction error needs to be saved by the FFV1 codec with a certain value of bits (due to distribution of probability of occurrence of all values in image). In fact, it is possible to do not use and save this 0 error for each fourth pixel, but in this case, the special, untypical codec need to be designed. However such codec will not be universal for the other future formats like e.g., RYYC and RYYB.

If we take under analysis the strategy with all four components (R, C, C, C) separated, we see higher compression ratios and throughputs than in previous strategies. Inside this procedure the compression of R component (${\mathrm{CR}}_{R}$) is slightly worse than the compression of C components (${\mathrm{CR}}_{C1}$, ${\mathrm{CR}}_{C2}$, ${\mathrm{CR}}_{C3}$). This strategy of decomposition reaches the very best decompression throughputs (${\mathrm{Thr}}_{\mathrm{out}}=81.8\;\mathrm{MB}/s;\;46\;\mathrm{fps}$ for the extended mode of compression/decompression).

Generally, the decompression throughputs for the extended mode are significantly lower (${\mathrm{Thr}}_{\mathrm{out}}$ ranging from 29 to 46 fps for the best cases) than for the standard mode (${\mathrm{Thr}}_{\mathrm{out}}$ ranging from 74 to 75 fps) (about 2.5 times lower). It is mainly due to the limitations of the possibility of parallelizing codec procedures.

Beside the decompression throughput, the best results, in all other indicators, offers the compression of RCCC images decomposed into R, C1 and C2C3 components. This comes especially from the best prediction in case of C2C3 joined components (${\mathrm{CR}}_{C2C3}=2.21$ for GOP = 1 and ${\mathrm{CR}}_{C2C3}=2.30$ for GOP = 100).

Taking into account the performance, in general, the performance of encoding/decoding significantly depends on the achieved CR value (the higher CR, the lower amount of data to process).

Comparing two best strategies, i.e., that with all separated components and that comprising C2 and C3 components but separated C1 and R components, we notice that the C2 and C3 components, due to their direct proximity, are together more efficiently compressed than separately. Compression ratios are CR = 2.06 (for GOP = 1) and CR = 2.20 (for GOP = 100) for all components separated, and CR = 2.12 (for GOP = 1) and CR = 2.23 (for GOP = 100) for comprised C2 and C3 components.

The differences between compression rates for the same sequences bring us to our next observation regarding the method of compression. The GOP set to 1 enters the encoder into standard mode while GOP = 100 enters it into extended mode. In general, the extended mode gives slightly higher CR values and very similar ${\mathrm{Thr}}_{\mathrm{in}}$ (throughput of compression) in comparison to the standard mode. The throughput of decompression (${\mathrm{Thr}}_{\mathrm{out}}$) is much higher in the standard mode (this mode enables native parallelism of computations).

Finally, the strategy with comprised C2 and C3 components but separated C1 and R components seems to be the best for the considered lossless compression of the HDR RCCC ADAS/ADS images.

In case of the HDR RCCC format, regardless the strategy of the division of the image components, the obtained compression ratios are much lower than that stated in Section 4, i.e., around 3.3 for 24 bpp RGB images (with 8 bit per one color component). This is due to several reasons:

- The codec is designed and optimized for typical color images, i.e., RGGB, not RCCC. The misprediction rate during the compression is higher.
- In RGGB with 3 × 8 bit per pixel, there is only one source component, i.e., R, G, or B for one pixel (cf. Figure 2b). To obtain the full RGB pixel, two remaining components are to be interpolated. Interpolation means that there is no additional source information. Therefore the prediction is better, and in consequence the compression ratio, is higher. In the RCCC format each pixel carries independently measured information and the correlation between values is much smaller than in the case of the RGGB format.
- Good quality 8-bit per component images contain typically less noise than HDR ones. Due to the unpredictable noise, the prediction during the image compression is much less accurate. To prove it we additionally performed a special test for 8 bpp SDR RCCC images. The SDR RCCC images were obtained from the 12-bit PWL bit-length compressed HDR source sequences by removing the least four bits. In this case we achieved average value of CR equal to 3.4, which is similar to 3.3, reported in [15]. This shows that even for untypical RCCC format it is possible to achieve high CR values, comparable to the potential of FFV1 codec. On the other hand, the much lower values of CR for 12 bpp RCCC images confirm that the last four bits in the test recordings are really noised.

### 6.6. Efficient Implementation of the Best Lossless HDR RCCC Codec Procedure

As it was mentioned in the previous subsection, the best proposed strategy is to divide the RCCC image into three sub-images: R, C1, and C2C3. In our case this means that the source RCCC image, e.g., with the resolution of 1280 × 969 pixels, is divided: into two images (those for R and C1 components) with the resolution of 640 × 485 pixels (i.e., with both horizontal and vertical source dimensions divided by 2) and to one joint C2C3 image with the resolution of 1280 × 484 pixels (i.e., with the vertical dimension only divided by 2).

In order to make the coder computationally efficient and to achieve real-time processing on a single CPU, the coding procedure needs to be multithreaded. Therefore, in the next step the separated R, C1, and C2C3 sub-images are compressed in parallel (in separated threads) by the lossless video codec (FFV1 codec was used in the experiments) and then written to a video container as three separated streams.

**Figure 7:**
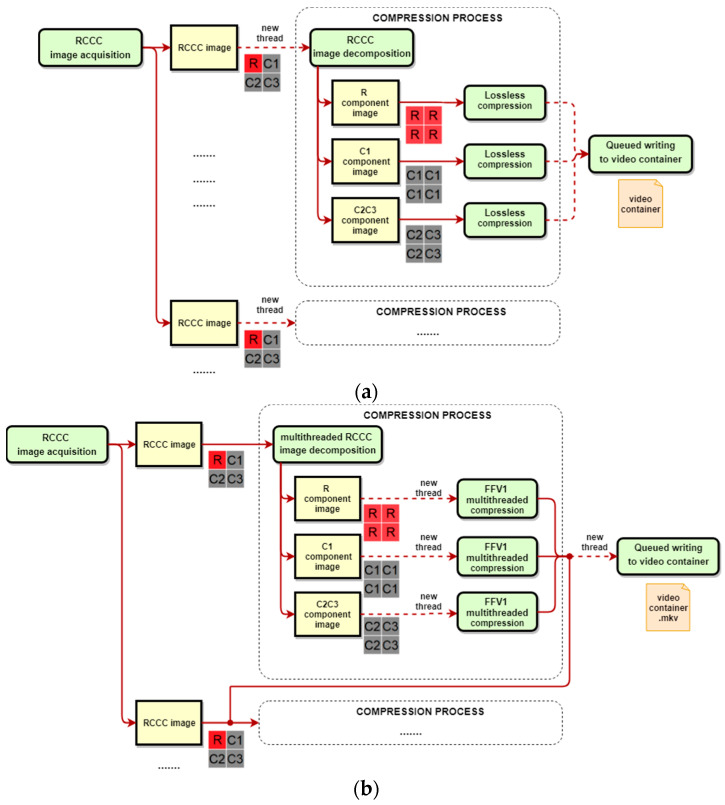
RCCC lossless compression procedures with FFV1 codec for separated R, C1 and joined C2 and C3 components in: (**a**) intra-frame mode, (**b**) inter-frame mode.

To speed-up the processing, the compression process is parallelized by starting new compression thread with a new RCCC image (or more precisely with three new images, i.e., R, C1, C2C3). Only the writing procedure to the common video container needs to be queued. A scheme of the whole proposed procedure is presented in Figure 7a. It is assumed that the FFV1 codec works in the standard mode.

Taking into account the priority of a highest possible compression ratio for ADAS/ADS applications, we rather prefer to use FFV1 codec in the highest compression mode, i.e., the extended mode with high GOP values (e.g., GOP = 100, cf. Table 2 and Table 3). However, to achieve the required compression efficiency, i.e., real time operation, it is necessary to adapt the RCCC codec procedure as it is not possible to perform parallel processing simply by running a new compression thread for each frame as it was done in the standard mode. The process of dividing the RCCC image into component images (or joining component images into one RCCC image in the decompression process) is now performed with multithreaded manner, to minimize loss of performance. The compression (or decompression) of component images is performed in parallel and multithreaded way for each component image to achieve full CPU usage. Finally, the writing and reading steps is performed in the new separated thread for each compressed image (and queued) to avoid suspending of the compression process of the next image frame.

The final procedure that effectively supports the extended mode of the FFV1 codec is presented in Figure 7b.

### 6.7. Detailed Tests of the Final Implementation of Lossless HDR RCCC Codec Procedure

In the last bunch of experiments we tested in detail the final implementation of the lossless HDR RCCC codec procedure with the best decomposition mode, i.e., this with R, C1 and C2C3 components. In these experiments we tested our procedure with all the HDR RCCC sequences separately.

Results are presented in Table 4. They show that achieved CR values (ranging from 1.69 to 2.16 for standard mode and from 1.81 to 2.43, for extended mode) depend on the image contents, quality, codec performance and the specificity of the RCCC format. Beside the video sequence number 3 (test ride in laboratory, inside the building, somehow synthetic case) the compression ratios (2.02≤CR≤2.43) and compression throughputs ($61\;\mathrm{MB}/s\leq {\mathrm{Thr}}_{\mathrm{in}}\leq 71\;\mathrm{MB}/s$) are similar, regardless the compression mode (standard or extended).

While the achieved values of ${\mathrm{Thr}}_{\mathrm{in}}$ for both compression modes are very similar (in the best cases: 71–72 fps), the decompression process performs slower for the extended mode. This is caused by a slower decompression speed of FFV1 codec than during the compression, for the same codec parameters and settings of multithread processing.

In fact, the throughputs reach up to 72 fps for compression process and 81 fps for decompression process. Comparing these results to the real-time input stream which has 36 fps, we may say that the real-time efficiency is achieved on one mid-class CPU (even with big surplus). Two things contributed to this result: the relatively efficient FFV1 codec and the carefully designed multithreaded programming procedure.

## 7. Conclusions

Up to now, special applications in the automotive research require lossless video compression for ADAS and ADS applications, so any loss of data during the compression is unacceptable. Therefore, in this paper we presented and tested the highly efficient lossless video compression procedure, along with its almost equally effective variants, according to the HDR RCCC standard (which is now becoming popular in ADAS and ADS applications), using for experiments the lossless FFV1 codec. All proposed procedures meet two strong requirements: real-time processing and high compression ratio.

We showed that compression of separated components is significantly more efficient than direct compression of RCCC images. We considered four possibilities and showed that the proper selection of the image decomposition strategy helps to increase the compression ratio and the overall performance of the compression and decompression. The best strategy, consisting in division of the original RCCC image into three sub-images: R, C1, and C2C3, allows to achieve high performance (up to 72 fps for compression and up to 81 fps for decompression performed using a single CPU).

Our experiments showed that even for deep 12 bpp HDR RCCC image format, the achievable compression ratios are still relatively high (ranging from 1.81 to 2.43, for standard mode). The achieved compression ratios depend on the image content, quality, codec performance, and preparation of the RCCC format for the compression.

## Figures and Tables

**Table 1:** Description of tested video sequences of RCCC format.

Sequence Number	Number of Frames	Description
1	1855	urban, sunny
2	1875	urban, sunny
3	515	test ride in laboratory
4	2011	urban, sunny
5	2051	road crossing, winter
6	2114	Suburban, cloudy
7	1761	road crossing, sunny
8	1707	suburban, evening
9	1909	suburban, sunny
10	1897	departure from the property
11	2149	road crossing, cloudy
Total frames	19,844	

**Table 2:** Average compression ratios for various lossless coding procedures and division of components.

Compression Mode	GOP	Coding Procedure	CR	${\mathrm{CR}}_{R}$	${\mathrm{CR}}_{\mathrm{CCCC}}$	${\mathrm{CR}}_{C1}$	${\mathrm{CR}}_{C2}$	${\mathrm{CR}}_{C3}$	${\mathrm{CR}}_{C2C3}$
standard	1	Direct—RCCC	1.47	-	-	-	-	-	-
standard	1	R images and interpolated CCCC	1.97	1.98	1.96	-	-	-	-
standard	1	All four components (R, C, C, C) separated	2.06	1.98	-	2.08	2.08	2.08	-
standard	1	Separated R, $C_{1}$ and joined $C_{2}C_{3}$ components	2.12	1.98	-	2.08	-	-	2.21
extended	100	Direct—RCCC	1.50	-	-	-	-	-	-
extended	100	R images and interpolated CCCC	2.04	2.12	2.01	-	-	-	-
extended	100	All four components (R, C, C, C) separated	2.20	2.12	-	2.21	2.21	2.23	-
extended	100	Separated R, $C_{1}$ and joined $C_{2}C_{3}$ components	2.23	2.12	-	2.21	-	-	2.30

**Table 3:** Throughputs for various lossless coding procedures and compression modes.

	Compression Mode							
Coding Procedure	Standard (GOP = 1)				Extended (GOP = 100)			
	$\mathrm{Compression}\;{\mathrm{Thr}}_{\mathrm{in}}$		$\mathrm{Decompression}\;{\mathrm{Thr}}_{\mathrm{out}}$		$\mathrm{Compression}\;{\mathrm{Thr}}_{\mathrm{in}}$		$\mathrm{Decompression}\;{\mathrm{Thr}}_{\mathrm{out}}$	
	[MB/s]	[fps]	[MB/s]	[fps]	[MB/s]	[fps]	[MB/s]	[fps]
Direct—RCCC	73.6	41	77.3	44	72.8	41	16.6	9
R images and interpolated CCCC	103.1	58	124.8	70	102.7	58	30.2	17
Separated all components	115.9	65	130.9	74	116.3	66	81.8	46
Separated R, $C_{1}$ and $C_{2}C_{3}$ component images	116.6	66	132.7	75	116.2	65	50.6	29

**Table 4:** Compression ratios and throughputs in the best lossless coding procedure (with separation into R, C1, C2C3 components) for various video HDR RCCC sequences and compression modes.

	Compression Mode									
Sequence	Standard (GOP = 1)					Extended (GOP = 100)				
	Compression CR and ${\mathrm{Thr}}_{\mathrm{in}}$			$\mathrm{Decompression}\;{\mathrm{Thr}}_{\mathrm{out}}$		$\mathrm{Compression}\;\mathrm{CR}\;\mathrm{and}\;{\mathrm{Thr}}_{\mathrm{in}}$			$\mathrm{Decompression}\;{\mathrm{Thr}}_{\mathrm{out}}$	
	CR	[MB/s]	[fps]	[MB/s]	[fps]	CR	[MB/s]	[fps]	[MB/s]	[fps]
1	2.02	110.9	63	127.3	72	2.14	110.7	62	51.1	29
2	2.15	114.2	64	135.2	76	2.27	112.8	64	55.2	31
3	1.69	96.9	55	110.9	62	1.81	98.3	55	42.4	24
4	2.27	117.9	66	139.7	79	2.38	117.3	66	52.6	30
5	2.31	125.1	70	141.7	80	2.42	124.1	70	50.9	29
6	2.33	127.4	72	143.1	81	2.43	126.1	71	49.8	28
7	2.05	116.1	65	130.7	74	2.18	115.6	65	50.5	28
8	1.91	108.5	61	122.5	69	2.03	108.9	61	48.6	27
9	2.13	119.4	67	132.1	74	2.24	119.5	67	53.5	30
10	2.16	120.3	68	134.5	76	2.27	120.1	68	52.7	30
11	2.31	125.6	71	142.1	80	2.42	124.3	70	49.9	28
Average	2.12	116.6	66	132.7	75	2.23	116.2	65	50.6	29

## Data Availability

3rd Party Data. Restrictions apply to the availability of these data. The data are not publicly available.
